# scReClassify: post hoc cell type classification of single-cell rNA-seq data

**DOI:** 10.1186/s12864-019-6305-x

**Published:** 2019-12-24

**Authors:** Taiyun Kim, Kitty Lo, Thomas A. Geddes, Hani Jieun Kim, Jean Yee Hwa Yang, Pengyi Yang

**Affiliations:** 1 School of Mathematics and Statistics, Faculty of Science, The University of Sydney, 2006 NSW, Australia; 2 Computational Systems Biology Group, Children’s Medical Research Institute, Faculty of Medicine and Health, The University of Sydney, 2145 NSW, Australia; 3 Charles Perkins Centre, The University of Sydney, 2006 NSW, Australia; 4 School of Life and Environmental Sciences, Faculty of Science, The University of Sydney, 2006 NSW, Australia

**Keywords:** Single-cell RNA-seq, scRNA-seq, Cell type classification, Class label noise

## Abstract

**Background:**

Single-cell RNA-sequencing (scRNA-seq) is a fast emerging technology allowing global transcriptome profiling on the single cell level. Cell type identification from scRNA-seq data is a critical task in a variety of research such as developmental biology, cell reprogramming, and cancers. Typically, cell type identification relies on human inspection using a combination of prior biological knowledge (e.g. marker genes and morphology) and computational techniques (e.g. PCA and clustering). Due to the incompleteness of our current knowledge and the subjectivity involved in this process, a small amount of cells may be subject to mislabelling.

**Results:**

Here, we propose a semi-supervised learning framework, named scReClassify, for ‘post hoc’ cell type identification from scRNA-seq datasets. Starting from an initial cell type annotation with potentially mislabelled cells, scReClassify first performs dimension reduction using PCA and next applies a semi-supervised learning method to learn and subsequently reclassify cells that are likely mislabelled initially to the most probable cell types. By using both simulated and real-world experimental datasets that profiled various tissues and biological systems, we demonstrate that scReClassify is able to accurately identify and reclassify misclassified cells to their correct cell types.

**Conclusions:**

scReClassify can be used for scRNA-seq data as a post hoc cell type classification tool to fine-tune cell type annotations generated by any cell type classification procedure. It is implemented as an R package and is freely available from https://github.com/SydneyBioX/scReClassify

## Background

Accurate identification of cell types from complex tissues across different organisms is critical for research in developmental biology [1] and cell reprogramming [2]. It is also an essential step towards a comprehensive understanding of the formation of complex organs such as heart [3], brain [4], and liver [5], and various cancers [6, 7]. Single-cell RNA sequencing (scRNA-seq), a technique that allows the transcriptomes of individual cells to be quantified, has become the key enabling technology for profiling cell types [8]. Significant effort and resources have been devoted for cell type profiling, such as the building of the mouse cell atlas [9] and the ongoing effort to generate the human cell atlas [10], but the accurate identification of the cell types based on scRNA-seq data remains a significant challenge [11].

Typically, cell types can be identified from scRNA-seq data by human inspection using a combination of prior biological knowledge such as marker genes, cell type specific characteristics such as morphologies, physiologies and functions [12, 13] and visualisation and unsupervised computational techniques such as principal component analysis (PCA), t-distributed stochastic neighbor embedding (tSNE) [14], self-organizing maps (SOMs) [15], and various clustering approaches [11, 16, 17]. Although these approaches can often lead to the correct identification of the majority of the cells in a scRNA-seq experiment, a small amount of cells may be mislabelled due to the incompleteness of our current knowledge and the subjectivity involved in the process [18, 19].

More recently, various supervised learning methods have been proposed for classifying cell types based on large collections of cell atlases [20, 21], mapping cell types [22, 23] and subtypes [24] across multiple scRNA-seq datasets, and predicting cell types in a scRNA-seq dataset by transfer learning from multiple related scRNA-seq datasets [25]. While these developments create new avenues for cell type identification, all these methods assume the cell type annotation in the training dataset is completely and perfectly defined beforehand, and therefore, their performance may suffer when mislabelled cells are present in the training dataset.

**Fig. 1:**
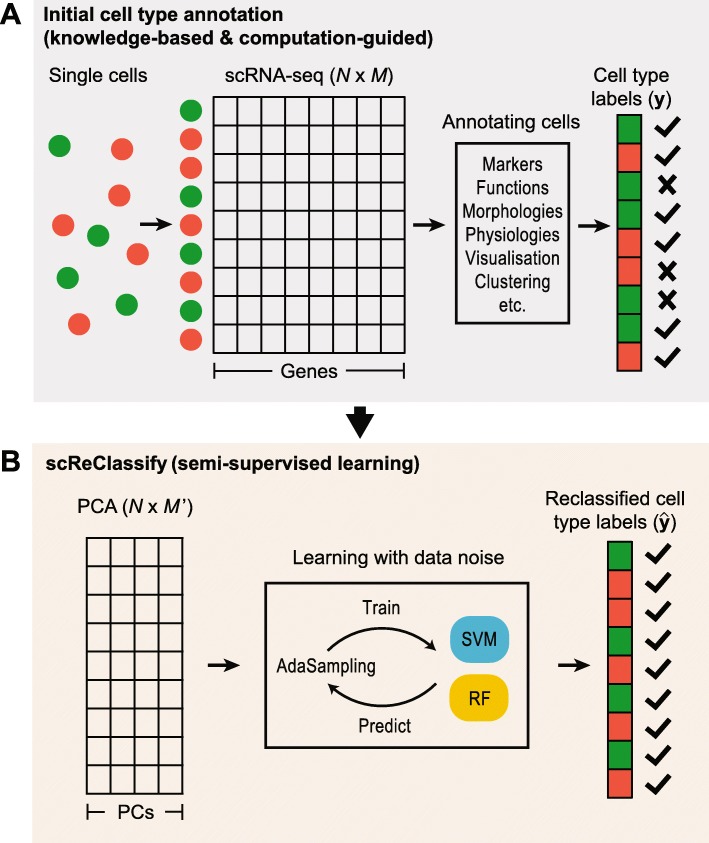
An illustration of the scReClassify framework. **a** The initial cell type annotation. This is typically achieved by using a combination of biological knowledge and computational approach. **b** Correction of mislabelled cells using an AdaSampling procedure. The dimensionality of the gene expression matrix is first reduced by PCA and mislabelled cells are identified and reclassified to their correct cell types by using AdaSampling with either a support vector machine (SVM) or a random forest (RF) classifier or an ensemble of SVMs or RFs

In this paper, we propose a post hoc cell type identification procedure to correct for any potentially mislabelled cells in a scRNA-seq dataset. To achieve this, we formulate the task of cell type identification as a machine learning problem in the presence of label noise [26], and apply a semi-supervised learning framework (named scReClassify) to identify potentially mislabelled cells and subsequently reclassify them to their correct cell types (Fig. 1). Specifically, scReClassify first reduces the dimensionality of scRNA-seq gene expression data into principal components (PCs) and subsequently applies a soft-label learning step, an extension to our recently proposed AdaSampling procedure [27], to create a model using the initial cell type annotations and reclassifies potentially mislabelled cells into their correct categories.

We evaluate the performance of scReClassify using both simulated datasets as well as real-world experimental datasets encompassing various tissues and biological systems. Our benchmark experiments and case studies demonstrate that scReClassify is able to accurately identify mislabelled cells in scRNA-seq datasets and reclassify them to their correct cell types. We envisage scReClassify to be used as a post hoc cell type classification tool, after standard cell type identification procedure has been performed, to fine-tune cell type annotations in scRNA-seq data analysis.

## Results

### Evaluation of scReClassify on simulated datasets

**Fig. 2:**
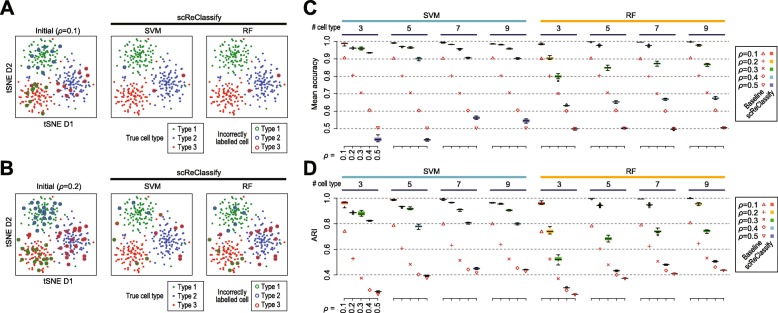
Evaluation of scReClassify on simulated datasets. In (**a**) and (**b**), each cell is shown as a solid point on each tSNE plot and coloured coded by its true cell type. The initial cell type labels (left panels in A and B) are the true cell types but with either 10% (**a**) or 20% (**b**) mislabelling. The solid point with an open ring represent incorrectly label cells and the ring colour represent the mislabelled type. In (**c**) and (**d**), scRNA-seq with varying percentages of mislabelled cells were simulated. Baselines represent the (**c**) mean accuracy and (**d**) adjusted Rand index (ARI) calculated from the ground truth and the initial annotations. Performance of scReClassify are shown as boxes coloured according to the percentages of mislabelled cells

To study the behaviour of scReClassify in learning from and correcting the initial cell type labels, we first tested its performance on synthetic scRNA-seq datasets with 10% or 20% mislabelled cells (i.e. *ρ*=0.1 or 0.2). The left panels in Fig. 2a and b visualise the true cell types and the mislabelled cells in the initial cell type labels in the two simulated datasets. After applying scReClassify with either SVM or RF as the base classifier, most of the mislabelled cells were corrected (accuracy up to 99%) with very few remaining mislabelled cells.

Unsurprisingly, the performance of scReClassify is affected by the quality of the initial cell type labels and a higher mislabelling percentage in the initial annotation is likely to result in more uncorrected cells even after applying scReClassify (Fig. 2). To investigate the relationship between the percentage of mislabelling in the initial annotation and the performance of scReClassify, we varied the range of *ρ* from 0.1 to 0.5 and assessed the performance of scReClassify on label correction of mislabelled cells using both mean classification accuracy and ARI (Fig. 2c and d). We found that in most cases scReClassify resulted in less mislabelled cells when *ρ* was set to less than or equal to 0.4 and, unsurprisingly, scReClassify was unable to improve cell type labels when half of the cells were mislabelled in their initial annotation (*ρ*=0.5). In particular, scReClassify with SVM showed notably better performance than with RF according to both mean accuracy (Fig. 2c) and ARI (Fig. 2d). These results suggest that RF in comparison to SVM may be more susceptible to overfitting and therefore leads to less efficient correction of mislabelled cells. Overall, scReClassify shows a robust performance when the level of mislabelled cells is low, and this is particularly apparent when *ρ*≤ 0.4 and SVM was used as the base classifier (Fig. 2c).

### Determining ensemble size

**Fig. 3:**
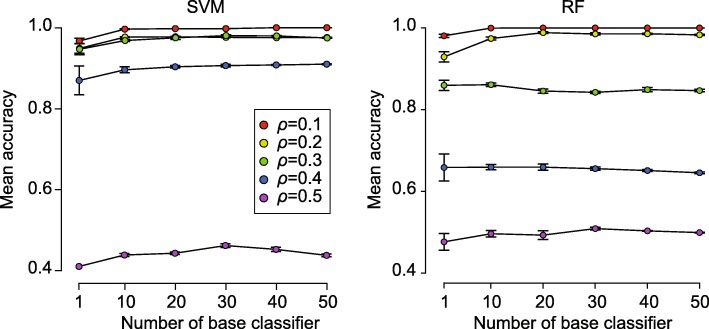
Ensemble size of scReClassify. The x-axis shows the number of base classifiers used to form the ensemble in scReClassify. Each line shows the mean cell type classification accuracy under different levels of call type label noise and using different ensemble sizes

One of the key parameters that may affect the performance of scReClassify is the number of base classifiers used to form the ensemble. To determine the minimum ensemble size required to achieve sufficient performance, we varied the number of base classifiers used to form the ensemble in scReClassify from 10 to 50 with a step of 10 and evaluated their performance using simulated scRNA-seq datasets with five cell types and *ρ* ranged from 0.1 to 0.5 (Fig. 3). We found the ensemble models of SVM and RF were better than their singles (i.e. ensemble size of 1) when the noise ratio was small *ρ*≤ 0.2). However, only SVM maintained significant improvements when *ρ* increased to 0.3 and 0.4. Overall, the improvement of ensemble models over their respective single model was mild and an ensemble size of 10 was sufficient for achieving desirable performance of scReClassify.

### Evaluation of scReClassify on experimental datasets

To test if scReClassify can correctly reclassify mislabelled cells in real-world scRNA-seq datasets generated from diverse biological systems, we introduced different proportions of mislabelled cells (*ρ* range from 0.1 to 0.5), as was done for the simulated datasets, to each of the four experimental datasets as detailed in Table 1.

**Table 1:** Summary of experimental scRNA-seq datasets used for method evaluation

ID	Publication	Description	Organism	# cell	# class	Protocol
E-MTAB-3929	[1]	Early human development	Human	1059	3	SMART-Seq2
GSE87795	[5]	Fetal liver development	Mouse	367	6	SMARTer
GSE60361	[4]	Cortex and hippocampus	Mouse	3005	7	SMARTer
GSE82187	[28]	Striatum	Mouse	705	10	SMARTer

**Fig. 4:**
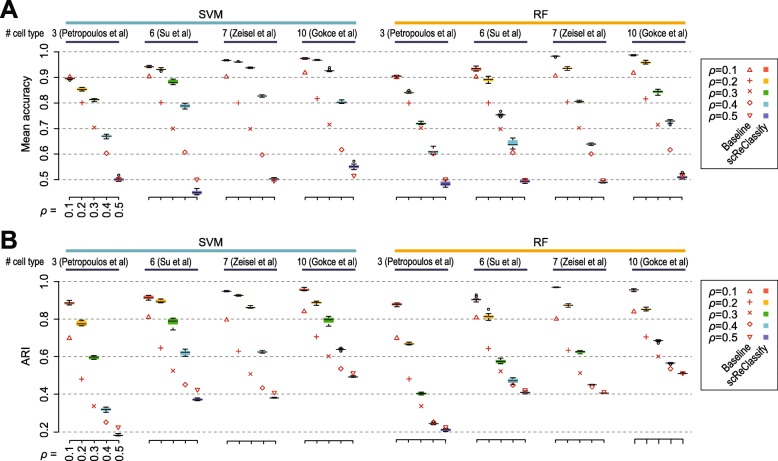
Evaluation of scReClassify on experimental datasets. Varying percentages of mislabelled cells were introduced to each scRNA-seq dataset to create an initial cell type annotation with different level of label noise (*ρ* range from 0.1 to 0.5). The performance in terms of mean accuracy (**a**) and ARI (**b**) calculated from the gold standard cell type annotation (annotation of each dataset from its original studies) and the initial cell type annotation (baseline), and the scReClassify corrected cell type annotation. scReClassify was repeated 10 times to capture the variability and shown as boxes coloured according to the percentages of mislabelled cells

We found that in most cases scReClassify with either SVM or RF reduced the number of mislabelled cells. However, the improvements in terms of both mean accuracy and ARI are lower than those observed in simulations (Fig. 4). One possible explanation is that there may be a proportion of mislabelled cells in each of four real-world experimental datasets even before the post hoc introduction of mislabelled cells. Furthermore, the performance of scReClassify also appears to be dataset dependent. For example, the reductions on mislabelled cells in the fetal liver development dataset [5] and in datasets that profiles different types of brain tissues [4, 28] are relatively higher than that in early human development dataset [1]. This may be due to the biological signal-to-noise ratios and unique structural properties inherent in each dataset. Consistent with the simulation results, scReClassify with SVM shows better performance than those from using RF. Also in agreement with the simulation results, a clear reduction of mislabelled cells is achieved by scReClassify with SVM when the level of noise *ρ* is smaller or equal to 0.4, and scReClassify is unable to reduce the percentage of mislabelled cells when *ρ*=0.5.

Comparing the performance of scReClassify with baseline (calculated from the ground truth and the initial noisy cell type annotations) on the early human development dataset [1], it appears that at *ρ*=0.1 there is no improvement based on mean accuracy (Fig. 4a) but ARI indicates a clear improvement of cell type classification after applying scReClassify (Fig. 4b). This is because the mean accuracy metric does not take into consideration the size difference of the cell types (i.e. number of cells annotated as Epiblast, Endoderm, and Trophectoderm are 122, 105, and 832) whereas ARI account for such size differences among different cell types [29]. This demonstrate the importance to measure performance using different metrics. Together, these results suggest that scReClassify can recover mislabelled cells in both simulated datasets and real-world scRNA-seq datasets even when a large proportion of the cells are mislabelled initially.

### scReClassify identifies and corrects potentially misclassified cells in experimental datasets

Since the performance of scReClassify on real-world experimental datasets is lower than those from simulated datasets, we hypothesized that a small proportion of cells may be mislabelled in each of four real-world experimental datasets even before our post hoc introduction of mislabelled cells. We therefore applied scReClassify (using SVM as the base classifier) to correct the cell type annotation of each dataset obtained directly from their original study. This has led to the correction of 28 [1], 12 [5], 73 [4], and 9 [28] cells corresponding to 2.6%, 3.3%, 2.4%, and 1.3% of all cells in each of the four datasets.

**Fig. 5:**
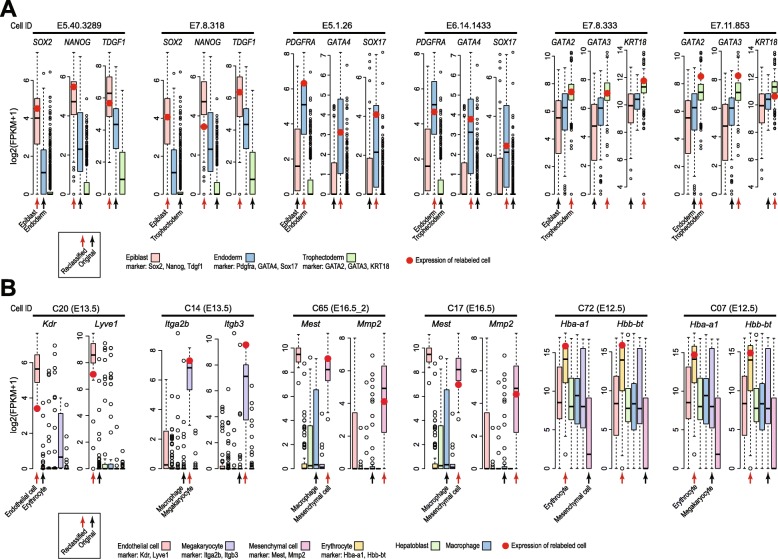
Comparison of gene expression levels of reclassified cells in cell type specific markers for early human development dataset (**a**) and fetal liver development dataset (**b**). The x-axis of the plots are grouped by cell types and the y-axis represent log2 transformed expression values. The boxes (coloured by cell types) represent expression distribution of corresponding cell type in the dataset. The red markers indicate the marker gene expression levels of the reclassified cell in the reassigned cell type. For each reclassified cell, the black arrows indicate the cell type annotation from its original publication and the red arrows indicate the newly scReClassify assigned cell type

We next performed case studies to validate some of the cells that are re-classified by scReClassify to a different cell type from their original annotation. Specifically, for the early human development dataset, we defined three marker genes for each of the three cell types in the dataset based on literature. These are Sox2, Nanog, and Tdgf1 for cells from Epiblast; Pdgfra, Gata4, and Sox17 for cells from Endoderm; and Gata2, Gata3, and Krt18 for cells from Trophectoderm. Figure 5a highlights six cells that represent all possible combinations of re-classification in which cells that are originally labeled as Endoderm (E5.40.3289) or Trophectoderm (E7.8.318) and are re-classified as Epiblast; cells that are originally labeled as Epiblast (E5.1.26) or Trophectoderm (E6.14.1433) and are re-classified as Endoderm; and cells that are originally labeled as Epiblast (E7.8.333) or Endoderm (E7.11.853) and are re-classified as Trophectoderm. As can be seen from Fig. 5a, the re-classified cell types fit significantly better to the marker genes than the originally assigned cell type for all six cells. Similarly, Fig. 5b highlights six cells from four cell types that are re-classified to a different cell type from their original cell type assignment in the fetal liver development dataset [5]. Again, the marker genes curated from literature clearly support these re-classification compared to their original cell type classification.

Together, these results indicate that a small proportion of cells are quite possibly misclassified in their original studies and scReClassify can serve as an effective tool to identify and reclassify these cells to their most likely cell type categories.

## Discussion

Cell type identification is an essential task in scRNA-seq data analysis. While a large number of methods have been proposed for achieving this goal, current methods typically rely on some form of prior biological knowledge, orthogonal biological data and/or computational techniques to assign cells to a set of possible cell types. These knowledge and empirical based approaches are likely to result in a small fraction of cells being incorrectly assigned to cell type. To fine-tune the initial annotations derived from experimental data, we proposed scReClassify, a post hoc cell type classification tool based on a semi-supervised learning approach. Taking the initial cell type annotations as soft-labels, it subsequently identifies and reclassifies potentially misclassified cells.

Current implementation of scReClassify does not take into consideration the sizes of different cell types in a scRNA-seq dataset. This is apparent when it is applied to early human development dataset [1]. While scReClassify did reduce the total number of misclassified cells according on ARI (Fig. 4b), the reduction on average mislabelling rate across each cell type is less drastic. One future extension of scReClassify is to account for the unequal size of cell types which in supervised learning is known as imbalanced classification problem [30]. To this end, different sampling and cost-sensitive learning methods could be considered. Another related extension of scReClassify is to estimate and account for different percentage of mislabelled cells. That is, the percentage of mislabelled cells in each cell type may be different from other cell types in a scRNA-seq dataset. Therefore, the sampling probabilities applied to each cell type may need to account for such differences in order to overcome classification bias towards cell types that are large and with lower proportion of mislabelled cells.

In scRNA-seq experiments, rare cell types with subtle difference in their transcriptome profiles are more likely to be mislabelled than large cell types with distinctive difference in their transcriptome profiles in comparison to other cell types. Moreover, cell types that are closely related to other and located in the ambiguous regions of the decision boundary are more likely to be mislabelled. The current model does not account for such a nonrandomness in cell type mislabelling. Modelling the nonrandom mislabelling in the initial cell type annotations may lead to further improvement of scReClassify performance. In addition, we found the performance of scReClassify scales well with different numbers of cell types, ranging from 3 to 10. Yet, given that large scRNA-seq datasets with considerably more cell types are increasingly common (such as the mouse and human cell atlas initiatives), it will be useful to assess the scalability of scReClassify on datasets with significantly more cells and much larger number of cell types.

While our application to the four experimental scRNA-seq datasets show reclassification of a relatively small percentage of cells from their original annotations, suggesting that the original cell type annotations are mostly high quality, our evaluation experiments on both simulation and these four real-world datasets demonstrate that scReClassify is robust even when the noise level is significantly higher. Therefore, the utility of scReClassify could become more apparent when the original annotation less from perfect.

## Conclusion

In summary, we have implemented a post hoc classification tool, scReClassify, to fine-tune cell type annotations generated using any methods in scRNA-seq datasets. As a simple, easy to use R package, we hope scReClassify will be a useful addition to the scRNA-seq data analysis toolkit.

## Methods

### The scReClassify framework

Here we summarise the proposed framework of scReClassify. Briefly, scReClassify (Fig. 1) expects an *N*×*M* expression matrix (denoted as *X*) with *N* being the number of cells and *M* being the number of genes. Importantly, it also expects that an initial cell type annotation of cells (denoted as **y**) is available. This initial cell type annotation may be inferred using prior biological knowledge such as cell functions, morphologies, physiologies and marker genes, and computational techniques such as PCA, tSNE, clustering and SOMs, or combinations of these approaches. Assuming both *X* and **y** are given for a scRNA-seq dataset, scReClassify performs post hoc cell type classification by first using PCA (“Determining ensemble size” section) to reduce the dimensionality of the expression matrix and then applying a semi-supervised learning procedure, AdaSampling (“AdaSampling and ensemble learning” section), to learn and adjust cell type labels for cells that are likely to be mislabelled in the initial annotation.

### The PCA dimension reduction procedure

Due to the high feature-dimensionality (i.e. the large number of measured genes in each cell) and that only a small fraction of them are cell type-specific and therefore are informative for cell type identification, it is often necessary to apply dimension reduction techniques prior to downstream cell type identification and analysis [16].

Starting from the original scRNA-seq expression dataset, which we denote as an *N*×*M* matrix as previously described, we apply PCA to perform dimension reduction. We select the number of PCs to use (*M*^′^) by first determining *d*, the number of PCs required to capture at least 70% of overall variability in the dataset. If *d* falls outside of the range of 10 and 20, we set *M*^′^ to either be 10 or 20 (summarised in Eq. 1). After the PCA procedure, the dimension of our original expression matrix will be reduced to *N*×*M*^′^.

1 $$  M' = \left\{ \begin{array}{ll} 10, & d < 10 \\ d, & 10 \leq d \leq 20 \\ 20, & d > 20 \end{array} \right.  $$

### AdaSampling and ensemble learning

Most scRNA-seq studies annotate each cell profiled in the experiments by its most likely cell type, and classify all cells into a finite set of cell types. Due to the technical limitations and/or limitations in current biological knowledge, the cell type annotation for a given scRNA-seq dataset may contain a proportion of mislabelled cells (a.k.a label noise and denoted as *ρ*). AdaSampling is a wrapper approach that couples a weighted sampling procedure with a probabilistic classification model for learning and correcting mislabelled instances in a dataset [27, 31]. Here we extend AdaSampling for multi-class classification and tailor its application for scRNA-seq datasets.

Let us denote the initial observed cell type labels as *y*_*i*_=*k*, where *k*∈{1,...,*K*} and *K* is the total number of cell types in a dataset, and *i* denotes cell index, where *i*∈{1,...,*N*} and *N* is the total number of cells. In a multi-class classification problem (*K*≥ 3), the probability of a cell being mislabelled *ε*_*i*_ can be estimated by a probabilistic classification model as follows:

2 $$ \varepsilon_{i} = P(\hat{y}_{i} \neq k | \mathbf{\mathrm{x}}_{i},y_{i} = k, D_{\rho}) = 1 - P(\hat{y}_{i} = k | \mathbf{\mathrm{x}}_{i},y_{i} = k, D_{\rho})  $$

where $P(\hat {y}_{i} \neq k |\mathbf {\mathrm {x}}_{i},y_{i} = k, D_{\rho })$ is the probability of the *i*th cell with a pre-assigned type of *k* been classified as a cell type that is not *k* by a given classifier trained on the dataset *D*_*ρ*_. Here x_*i*_∈*X* is the expression profile of the *i*th cell.

To identify these mislabelled cells and subsequently reclassify them to their correct cell types, AdaSampling starts by treating all cells with uniform probability $\frac {1}{n}$ of being selected and training a given classification model on *D*_*ρ*_ that estimate the mislabelling probability of each cell using Eq. (2). Subsequently, AdaSampling applies a weighted sampling from *D*_*ρ*_ in which each cell *i* will have a probability of 1−*ε*_*i*_ to be included in the updated training data *D*_*ρ*,*l*_ (where *l*=1,...,*L* denotes number of iterations). In other words, cells that are likely to be mislabelled (i.e. those with large *ε*_*i*_) will have less chance to be included in next iteration of model training. This procedure iteratively improves the accuracy of the trained classification model by improving the quality of the cell labels included in training the model. Our previous evaluation suggests that only a few iteration is needed to achieve a good performance [31]. Our implemented package allows users to specify this parameter and a default of 3 iterations was used in this study.

The final estimation of mislabelling probability for each cell is *ε*_*i*,*L*_ (*i*=1,...,*N*). A *ε*_*i*,*L*_ weighted sampling from *D*_*ρ*_ can be applied to generate the final training dataset *D*_*ρ*,*L*+1_, and the classification model trained on *D*_*ρ*,*L*+1_ can be used to reclassify all cells in the original dataset *D*_*ρ*_. Alternatively, *ε*_*i*,*L*_ weighted sampling can be performed multiple times and will result in $D^{b}_{\rho,L+1}, (1,..., B)$ training datasets. Each of the dataset then can be used to train a base classification model. An ensemble classification of all cells in *D*_*ρ*_ can be obtained by combining the classification probabilities of each cell from each of the base models.

### Base classifiers

The AdaSampling framework, essentially a wrapper procedure, relies on base classification models to estimate the mislabelling probability of each cell in a scRNA-seq dataset. In this work, we used either support vector machine (SVM) [32] or random forest (RF) [33] as base classifiers to provide such probabilistic estimates, but any other classifiers capable of providing probabilistic estimates can be used. Specifically, for SVM, the prediction probability of each cell is estimated using Platt’s method [34] as follows:

$$ P(\hat{y} = k|\mathbf{\mathrm{x}},D) = \frac{1}{1 + \text{exp}(A \times f(\mathbf{\mathrm{x}}) + B)} $$

$$ f(\mathbf{\mathrm{x}}) = \beta + \sum_{\tau \in S} \alpha_{\tau} \text{exp}(-\gamma ||\mathbf{\mathrm{x}} - \mathbf{\mathrm{x}}_\tau||^{2}_{2}) $$

where *S* is the support vector set and *A* and *B* are parameters (estimated by maximum likelihood) of sigmoid link function that converts the output *f*(x) from the SVM into a probability where *α*_*τ*_ and *β* represent the coefficient and intercept term of a classification vector respectively, and exp($-\gamma ||x-x_{\tau }||^{2}_{2}$) represents radial basis kernel. Note that we used one-against-one approach for multi-class learning with SVM.

For RF, the prediction probability of each cell is estimated from a collection of trees as follows:

3 $$ P(\hat{y} = k|\mathbf{\mathrm{x}},D) = \frac{1}{B}\sum_{b=1}^{B} \text{round}(h(\mathbf{\mathrm{x}}|\theta_{b}))  $$

where *h*(**x**|*θ*_*b*_) denote classification tree, each parameterised by *θ*_*b*_ by training on the *b*th bootstrap data sample and a subset of features randomly sampled from *M*^′^. The round(.) function converts the probability value to the nearest integer to either 0 or 1. We estimate the probabilities by making a class prediction for each tree, and counting the fraction of trees that vote for a certain class. *B* is set to a default value of 100.

### Simulated and experimental scRNA-seq datasets

We used both simulated and real-world experimental scRNA-seq datasets to evaluate the proposed method. To simulate scRNA-seq datasets, we used the Splatter R package [35]. Specifically, datasets with 3, 5, 7 and 9 cell types were simulated. The number of cells in each type was set to 100, the number of genes in each transcriptome was set to 10,000 and the probability of a gene being differentially expressed was set to 0.1. To simulate mislabelling, cell type label of 10%, 20%, 30%, 40% and 50% of randomly selected cells in each cell type group were flipped to the next cell type group. This was done for all cell types in each dataset and therefore introduced an overall of 10%, 20%, 30%, 40% and 50% mislabelled cells in each dataset with known ground truth label.

For experimental scRNA-seq datasets (Table 1), the cell type annotation information from their original studies were treated as ‘gold standard’ for method evaluation. The same procedure, in which 10%, 20%, 30%, 40% and 50% of the cells randomly selected in each cell type group were flipped into the next cell type group, was used to introduce mislabelled cells in each dataset. Both simulated and experimental scRNA-seq datasets were in unit fragments per kilobase of transcript per million mapped reads (FPKM) and were converted into log2(FPKM+1). Genes with more than 80% of missing values across cells were removed. This is because that while typically all genes are included in a scRNA-seq dataset, only a subset of genes are expressed. Therefore, many of the genes are expected to have no quantification and removing non-expressed genes in a scRNA-seq dataset is a common pre-processing approach [36]. After filtering, these datasets were used to assess if and to what degree a method can correctly recover the mislabelled cells in each dataset. These datasets were used to assess if and to what degree a method can correctly recover the mislabelled cells in each dataset.

### Evaluation metrics

The mean classification accuracy and adjusted Rand index (ARI) were used to assess the performance of cell type identification from each dataset. For each cell *i* in a scRNA-seq dataset, let us denote the observed cell type label as *y*_*i*_ and the ground truth label (or gold standard label in the cases of experimental datasets) as *s*_*i*_, then the mean classification accuracy can be defined as follows:

4 $$ \mathrm{mean~accuracy} = \frac{1}{K} \sum_{k=1}^{K} \frac{1}{N_{k}}\sum_{s_{i} = k} I\left(\hat{y}_{i}=s_{i}\right)  $$

where $\hat {y}_{i}$ is the cell type given by the classification model, *k*∈{1,...,*K*} is the index of cell types in a dataset with *K* cell types, *I*(.) is the indicator function, and *N*_*k*_ is the number of cells the *k*th cell type.

ARI is a measure of the concordance between two lists of groupings [37] and is widely used for benchmark performance of clustering method for scRNA-seq data. One advantage of ARI over mean classification accuracy is that the size of each cell type (i.e. the number of cells in each cell type group) is adjusted for in ARI whereas the mean classification accuracy does not distinguish between the different numbers of cells across cell type groups. Let $y_{i}, \hat {y}_{i}$, and *s*_*i*_ be the lists of cell type labels for each cell in a scRNA-seq dataset, then for any given two lists we can define *a* the number of pairs of cells labelled as the same type in both lists, *b* the number of pairs of cells labelled as the same cell type in the first list but as different cell types in the second list, *c* the number of pairs of cells labelled as different cell types in the first list but as the same cell type in the second list, and *d* the number of pairs of cells labelled as from different cell types in both the first and the second list. ARI is then defined as follows:

5 $$ \text{ARI}=\frac{2(ad-bc)}{(a+b)(b+d)+(a+c)(c+d)}  $$

## Data Availability

The datasets generated and/or analysed during the current study are available from the GEO, EBI, and Broad repositories. Details of datasets are listed in Table 1.

## Abbreviations

ARI: adjusted rand index
FPKM: fragments per kilobase of transcript per million mapped reads PCA: principal component analysis
scRNA-seq: Single-cell RNA-seq
SOMs: self-organizing maps
tSNE: t-distributed stochastic neighbour embedding

## References

[1] Petropoulos S, Edsgärd D, Reinius B, Deng Q, Panula SP, Codeluppi S, Reyes AP, Linnarsson S, Sandberg R, Lanner F (2016). Single-cell rna-seq reveals lineage and x chromosome dynamics in human preimplantation embryos. Cell.

[2] Nguyen Q, Lukowski S, Chiu H, Senabouth A, Bruxner T, Christ A, Palpant N, Powell J (2018). Single-cell rna-seq of human induced pluripotent stem cells reveals cellular heterogeneity and cell state transitions between subpopulations. Genome Res.

[3] DeLaughter DM, Bick AG, Wakimoto H, McKean D, Gorham JM, Kathiriya IS, Hinson JT, Homsy J, Gray J, Pu W (2016). Single-cell resolution of temporal gene expression during heart development. Dev cell.

[4] Zeisel A, Muñoz-Manchado AB, Codeluppi S, Lönnerberg P, La Manno G, Juréus A, Marques S, Munguba H, He L, Betsholtz C (2015). Cell types in the mouse cortex and hippocampus revealed by single-cell rna-seq. Science.

[5] Su X, Shi Y, Zou X, Lu Z-N, Xie G, Yang JY, Wu C-C, Cui X-F, He K-Y, Luo Q (2017). Single-cell rna-seq analysis reveals dynamic trajectories during mouse liver development. BMC Genomics.

[6] Puram SV, Tirosh I, Parikh AS, Patel AP, Yizhak K, Gillespie S, Rodman C, Luo CL, Mroz EA, Emerick KS (2017). Single-cell transcriptomic analysis of primary and metastatic tumor ecosystems in head and neck cancer. Cell.

[7] Zheng C, Zheng L, Yoo J-K, Guo H, Zhang Y, Guo X, Kang B, Hu R, Huang JY, Zhang Q (2017). Landscape of infiltrating t cells in liver cancer revealed by single-cell sequencing. Cell.

[8] Kolodziejczyk AA, Kim JK, Svensson V, Marioni JC, Teichmann SA (2015). The technology and biology of single-cell rna sequencing. Mol Cell.

[9] Han X, Wang R, Zhou Y, Fei L, Sun H, Lai S, Saadatpour A, Zhou Z, Chen H, Ye F (2018). Mapping the mouse cell atlas by microwell-seq. Cell.

[10] Rozenblatt-Rosen O, Stubbington MJ, Regev A, Teichmann SA (2017). The human cell atlas: from vision to reality. Nat News.

[11] Stegle O, Teichmann SA, Marioni JC (2015). Computational and analytical challenges in single-cell transcriptomics. Nat Rev Genet.

[12] Arendt D, Musser JM, Baker CV, Bergman A, Cepko C, Erwin DH, Pavlicev M, Schlosser G, Widder S, Laubichler MD (2016). The origin and evolution of cell types. Nat Rev Genet.

[13] Tirosh I, Venteicher AS, Hebert C, Escalante LE, Patel AP, Yizhak K, Fisher JM, Rodman C, Mount C, Filbin MG (2016). Single-cell rna-seq supports a developmental hierarchy in human oligodendroglioma. Nature.

[14] Maaten Lvd, Hinton G (2008). Visualizing data using t-sne. J Mach Learn Res.

[15] Kim DH, Marinov GK, Pepke S, Singer ZS, He P, Williams B, Schroth GP, Elowitz MB, Wold BJ (2015). Single-cell transcriptome analysis reveals dynamic changes in lncrna expression during reprogramming. Cell Stem Cell.

[16] Bacher R, Kendziorski C (2016). Design and computational analysis of single-cell rna-sequencing experiments. Genome Biol.

[17] Herring CA, Banerjee A, McKinley ET, Simmons AJ, Ping J, Roland JT, Franklin JL, Liu Q, Gerdes MJ, Coffey RJ (2018). Unsupervised trajectory analysis of single-cell rna-seq and imaging data reveals alternative tuft cell origins in the gut. Cell Syst.

[18] Grün D, van Oudenaarden A (2015). Design and analysis of single-cell sequencing experiments. Cell.

[19] Kim T, Chen IR, Lin Y, Wang AY-Y, Yang JYH, Yang P. Impact of similarity metrics on single-cell rna-seq data clustering. Brief Bioinformatics. 2018. 10.1093/bib/bby076. 10.1093/bib/bby076 30137247

[20] Xie P, Gao M, Wang C, Zhang J, Noel P, Yang C, Hoff DV, Han H, Zhang MQ, Lin W (2019). SuperCT: a supervised-learning framework for enhanced characterization of single-cell transcriptomic profiles. Nucleic Acids Res.

[21] Wagner F, Yanai I. Moana: A robust and scalable cell type classification framework for single-cell rna-seq data. bioRxiv. 2018:456129. 10.1101/456129.

[22] Kiselev VY, Yiu A, Hemberg M (2018). scmap: projection of single-cell rna-seq data across data sets. Nat Methods.

[23] Alquicira-Hernandez J, Nguyen Q, Powell JE. scpred: Single cell prediction using singular value decomposition and machine learning classification. bioRxiv. 2018:369538.

[24] Crow M, Paul A, Ballouz S, Huang ZJ, Gillis J (2018). Characterizing the replicability of cell types defined by single cell rna-sequencing data using metaneighbor. Nat Commun.

[25] Lieberman Y, Rokach L, Shay T (2018). Castle–classification of single cells by transfer learning: Harnessing the power of publicly available single cell rna sequencing experiments to annotate new experiments. PloS One.

[26] Frénay B, Verleysen M (2014). Classification in the presence of label noise: a survey. IEEE Trans Neural Netw Learn Syst.

[27] Yang P, Ormerod JT, Liu W, Ma C, Zomaya AY, Yang JY (2019). Adasampling for positive-unlabeled and label noise learning with bioinformatics applications. IEEE Trans Cybern.

[28] Gokce O, Stanley GM, Treutlein B, Neff NF, Camp JG, Malenka RC, Rothwell PE, Fuccillo MV, Südhof TC, Quake SR (2016). Cellular taxonomy of the mouse striatum as revealed by single-cell rna-seq. Cell Rep.

[29] Gates AJ, Ahn Y-Y (2017). The impact of random models on clustering similarity. J Mach Learn Res.

[30] Yang P, Yoo PD, Fernando J, Zhou BB, Zhang Z, Zomaya AY (2014). Sample subset optimization techniques for imbalanced and ensemble learning problems in bioinformatics applications. IEEE Trans Cybern.

[31] Yang P, Liu W, Yang J. Positive unlabeled learning via wrapper-based adaptive sampling. In: Proceedings of the Twenty-Sixth International Joint Conference on Artificial Intelligence, IJCAI-17. International Joint Conferences on Artificial Intelligence Organization: 2017. p. 3273–9. 10.24963/ijcai.2017/457.

[32] Cortes C, Vapnik V (1995). Support-vector networks. Mach Learn.

[33] Breiman L (2001). Random forests. Mach Learn.

[34] Platt J (2000). Probabilistic outputs for support vector machines and comparisons to regularized likelihood methods. Adv Large Margin Classif.

[35] Zappia L, Phipson B, Oshlack A (2017). Splatter: simulation of single-cell rna sequencing data. Genome Biol.

[36] Lin Y, Ghazanfar S, Wang KY, Gagnon-Bartsch JA, Lo KK, Su X, Han Z-G, Ormerod JT, Speed TP, Yang P (2019). scmerge leverages factor analysis, stable expression, and pseudoreplication to merge multiple single-cell rna-seq datasets. Proc Nat Acad Sci.

[37] Rand WM (1971). Objective criteria for the evaluation of clustering methods. J Am Stat Assoc.

